# Primary biliary cholangitis patients exhibit MRI changes in structure and function of interoceptive brain regions

**DOI:** 10.1371/journal.pone.0211906

**Published:** 2019-02-08

**Authors:** Victoria Mosher, Mark Swain, Jack Pang, Gilaad Kaplan, Keith Sharkey, Glenda MacQueen, Bradley Gordon Goodyear

**Affiliations:** 1 Department of Medicine, University of Calgary, Calgary, Alberta, Canada; 2 Seaman Family MR Research Centre, University of Calgary, Calgary, Alberta, Canada; 3 Snyder Institute for Chronic Diseases, University of Calgary, Calgary, Alberta, Canada; 4 Liver Unit – Calgary Division of Gastroenterology and Hepatology, University of Calgary, Calgary, Alberta, Canada; 5 Hotchkiss Brain Institute, University of Calgary, Calgary, Alberta, Canada; 6 Department of Physiology & Pharmacology, University of Calgary, Calgary, Alberta, Canada; 7 Department of Psychiatry, University of Calgary, Calgary, Alberta, Canada; 8 Mathison Centre for Mental Health Research and Education, University of Calgary, Calgary, Alberta, Canada; 9 Department of Radiology, University of Calgary, Calgary, Alberta, Canada; 10 Department of Clinical Neurosciences, University of Calgary, Calgary, Alberta, Canada; Texas A&M University, UNITED STATES

## Abstract

**Background:**

Many patients with primary biliary cholangitis (PBC) experience non-hepatic symptoms that are possibly linked to altered interoception, the sense of the body’s internal state. We used magnetic resonance imaging (MRI) to determine if PBC patients exhibit structural and functional changes of the thalamus and insula, brain regions that process signals related to interoception.

**Methods:**

Fifteen PBC patients with mild disease and 17 controls underwent 3 Tesla T_1_-weighted MRI, resting-state functional MRI, and quantitative susceptibility mapping (QSM), to measure thalamic and insular volume, neuronal activity and iron deposition, respectively. Group differences were assessed using analysis of covariance, and stepwise linear regression was used to determine the predictive power of clinical indicators of disease.

**Results:**

PBC patients exhibited reduced thalamic volume (*p* < 0.01), and ursodeoxycholic acid (UDCA) non-responders exhibited lower left thalamus activity (*p* = 0.05). PBC patients also exhibited reduced anterior insula activity (*p* = 0.012), and liver stiffness positively correlated with MRI indicators of anterior insula iron deposition (*p* < 0.02).

**Conclusions:**

PBC affects structure and function of brain regions critically important to interoception. Moreover, these brain changes occur in patients with early, milder disease and thus may potentially be reversible.

## Introduction

Primary Biliary Cholangitis (PBC) is an autoimmune liver disease characterized by destruction of the hepatic interlobular bile ducts. If left untreated, PBC can progress to cirrhosis, liver failure and transplantation or even death within 10 to 20 years [1]. Ursodeoxycholic acid (UDCA) can delay disease progression in some patients [2]; however, it does little to alleviate commonly reported behavioral symptoms [3–5] including fatigue [6–8], memory and concentration problems [9] and depressed mood [10, 11]. These symptoms significantly contribute to decreased quality of life [12], poor prognosis and increased mortality [13, 14], despite the fact that they appear unrelated to disease severity [7, 15].

Animal models of cholestatic liver disease strongly suggest that extra-hepatic symptoms have a neurological basis as a result of the impact of liver inflammatory responses (e.g., macrophage activation, cytokine production) on liver-to-brain signaling pathways (neural, cerebral endothelial cell and/or humoral pathways) [16]. Our recent study of PBC patients supports this assertion; using resting-state functional magnetic resonance imaging (fMRI), we observed altered functional connections of deep grey matter brain regions, in association with UDCA response, fatigue and verbal working memory performance [17]. One of those regions was the thalamus, which along with the insula, is thought to be involved in interoception. Interoception is the sense of the internal state of the body, and involves the brain’s process of integrating peripheral signals relayed from the body [18]. An fMRI study of unmedicated major depressive disorder (MDD) patients revealed abnormal interoceptive activity of the insula that was negatively correlated with depression scores and somatic symptom severity [19]. Another fMRI study observed increased activity of the thalamus and insula in response to a cognitive task in healthy controls given a typhoid vaccination (which initiates systemic inflammation that impairs mood, cognition and behavior), and inflammation-induced fatigue was predicted by activity within the mid/posterior insula [20]. These studies suggest that function (and possibly structure) of the thalamus and insula are strongly susceptible to altered interoception. Thus, it is plausible that the structure and function of the thalamus and insula are impacted in PBC patients as well, as a result of altered interoception in response to immune-mediated inflammation of the liver.

One way to non-invasively determine if liver inflammatory responses along liver-to-brain signaling pathways result in inflammation within the brains of PBC patients is by using quantitative susceptibility mapping (QSM). QSM imaging is a form of MRI that allows for the quantification of certain biomarkers of inflammation in the brain, such as iron, through a parameter known as susceptibility [21]. Iron within the brain is critical for the destruction of invading pathogens, as microglia/macrophages use iron to produce free radicals [22]. However, both a deficiency and a surplus of brain iron can be detrimental. It is hypothesized that disruption of iron metabolism may be responsible for iron accumulation in neurodegenerative diseases [23, 24] and may act as a biomarker of activation of the innate immune system within the brain [25]. In patients with multiple sclerosis (MS), increased iron deposition specifically within the thalamus has been reported [26, 27]. Further, using volumetric measurements from MRI, reduced thalamic volume has also been reported in MS patients, in correlation with decreased cognitive performance [28]. Thus, there appears to be an interrelationship between inflammation within the thalamus, thalamic volume reduction and patient behavior.

While our previous resting-state fMRI study demonstrated altered functional connections of the thalamus, it did not determine if the activity level of the thalamus itself was altered. This can be further inferred from resting-state fMRI data using an alternative analysis approach that determines the amplitude of low-frequency fluctuations (ALFF) of resting-state signals emanating from a brain region [29], which is thought to reflect the amplitude of spontaneous neural activity [30]. Changes in ALFF have been reported previously in a number of neurodegenerative diseases, and interestingly, MS patients exhibit alterations of ALFF of the thalamus [31, 32] and insula [31].

In the present study, we examined the thalamus and insula of the PBC patients of our previous study [17] from whom we obtained QSM data, and compared them to healthy control subjects. We also conducted additional analyses of our patient group to determine if volume, susceptibility or ALFF was associated with clinical indicators of disease, treatment response and symptom severity.

## Methods

### Participants

This study was approved by the Conjoint Health Research Ethics Board of the University of Calgary. Written informed consent was obtained from all participants prior to study participation. Of the participants in our previous study [17], we examined MRI data collected from 15 female patients with PBC (median age = 53 years, IQR = 9). Standard criteria for the diagnosis of PBC included anti-mitochondrial antibody positivity (AMA+) and abnormal cholestatic liver biochemistry prior to the initiation of UDCA. All patients were taking UDCA for at least six months (mean dose 15.0 mg/kg/day) (Table 1); complete UDCA response was defined as a sustained normalization of serum alkaline phosphatase levels after one year [33]. All patients were non-cirrhotic patients based on biochemistry (i.e., normal INR, and normal serum bilirubin and albumin concentrations) and liver stiffness (<16.9 kPa [34]), as measured by transient elastography (Fibroscan; Echosens, Paris, France). That is, only patients with clinically mild disease were included, to avoid potential issues associated with cirrhosis and hepatic encephalopathy. Total bilirubin level, alkaline phosphatase level, albumin and platelets after one year of UDCA therapy were used to compute GLOBE score, which predicts liver transplantation-free survival [35]. The PBC-40 questionnaire was completed by all PBC patients within 24 hours of MRI, to assess the impact of fatigue, pruritus and general symptoms, as well as emotional, cognitive and social function on quality of life [15, 36].

**Table 1:** Patient demographic and clinical characteristics.

Patient	Age (yrs)	Years since diagnosis	Fibroscan Value (kPa)	Alkaline Phosphatase (U/L)	Total PBC-40 Score	PBC-40 Pruritus Score	UDCA Responder (Y/N)	GLOBE score (including threshold)
1	52	2	3.0	162	41	0	Y	-1.63 (0.01)
2	38	2	6.9	132	52	0	N	-1.94 (-0.52)
3	60	8	4.0	121	65	5	Y	-0.11 (0.01)
4	72	8	3.7	251	45	5	N	no data
5	59	14	8.9	89	57	3	Y	no data
6	53	2	4.0	122	47	0	Y	-0.31 (0.01)
7	54	15	13.4	208	128	8	N	-1.02 (-0.52)
8	64	8	4.8	183	93	0	N	-1.44 (0.6)
9	60	4	4.3	154	64	3	N	-0.63 (0.6)
10	53	10	8.6	100	102	3	N	-0.81 (-0.52)
11	53	6	8.0	113	54	0	N	-0.33 (0.01)
12	68	9	4.8	147	70	1	N	-0.19 (1.01)
13	46	6	11.6	189	45	3	N	-0.95 (-0.52)
14	47	1	7.0	143	107	9	Y	-1.32 (0.01)
15	50	1	3.5	85	93	5	Y	-1.75 (0.01)

MRI data were also examined from 17 healthy female control subjects (age-matched as a group: median age = 53 years, IQR = 5). Exclusion criteria for all participants included significant medical comorbidities (diabetes, neurological disease, mood disorder, cardiac or respiratory disease), smokers, consumption of more than seven standard alcoholic drinks per week, or contraindications to MRI.

### MRI acquisition and processing

MRI was performed using a 3 Tesla GE Discovery MR750 scanner equipped with a 12-channel receive-only phased array head coil (GE Healthcare, Waukesha, WI). High-resolution T_1_-weighted anatomical images were collected using a three-dimensional magnetization-prepared rapid gradient echo sequence (MP-RAGE: inversion/repetition/echo time = 550/8.2/3.2 ms, 0.8x0.8x1.3 mm voxels). These anatomical images were processed for each participant using *FreeSurfer v6*.*0* to segment and calculate the volumes of the right and left thalamus [37]. Total intracranial volume was also determined. As *FreeSurfer* does not segment the insula, it was excluded from volumetric analysis.

QSM data were collected using an RF-spoiled, flow-compensated 3D gradient echo sequence (repetition/echo time = 29.5/26.3 ms; flip angle = 20°; FOV = 256x256x132 mm^3^; voxel size = 1x1x1 mm^3^; 8 echoes). QSM images were generated using *Cerebra-QSM* (Calgary Image Processing and Analysis Centre, Calgary, AB). QSM structural images were aligned to the T_1_-weighted anatomical images using the tools of *FSL* (http://www.fmrib.ox.ac.uk/fsl/). Average susceptibility was then computed for each of the left and right thalamus. Given the known differences in function of the anterior and posterior insula, the anatomical atlas tool of *FSL* was used to divide the insula into its anterior and posterior segments, and average susceptibility was computed for the left and right hemispheres.

A gradient-recalled echo, echo planar imaging sequence (repetition/echo time = 2500/30 ms, flip angle = 75°, 150 total volumes, 64 x 64 matrix, 3-mm isotropic voxels) was used to collect resting-state fMRI data. During data acquisition, participants were instructed to keep their eyes open and focused on a fixation cross [38]. Resting-state fMRI data underwent standardized pre-processing steps using *FSL*, including brain extraction, motion correction, intensity normalization, slice-timing correction, anatomical registration, 6-mm Gaussian kernel spatial smoothing and registration to anatomical images [39, 40]. ALFF was calculated for each image voxel by first transforming its time series into the frequency domain using the Fourier transform, calculating the power spectrum using the tools of *Matlab* (The Mathworks Inc., Natick, MA), and averaging power over the resting-state frequencies (0.01–0.1 Hz). Average ALFF for the thalamus, anterior and posterior insula were then computed for each of the left and right hemispheres.

### Data analysis

All data analyses were performed using *IBM SPSS Statistics for Macintosh*, *Version 24* (IBM Corp., Armonk, NY). For each of volume, susceptibility and ALFF, a repeated-measures (left and right hemisphere) analysis of covariance (ANCOVA) was used to examine group differences, with age as a covariate (total intracranial volume was an additional covariate for volumetric analyses). PBC-40 pruritus score was also used as a co-factor of interest, as pruritus could be associated with altered interoception (healthy control subjects were assigned an pruritus score of 0). A significance level of *p* = 0.05 was chosen to indicate a significant difference between groups.

Stepwise linear regression analyses in the forward direction were performed on PBC patient data of the left and right hemisphere, to examine the predictive power of Fibroscan value, years since diagnosis, alkaline phosphatase level, UDCA response (Y/N) and total PBC-40 score. A factor or combination of factors were deemed to be significant if the level of significance exceeded 95% (i.e., *p* < 0.05). It was found that all subscores of the PBC-40 were correlated with each other and with total PBC-40 score; hence, only total PBC-40 score was used in these analyses. It was also found that all patients were below the GLOBE score threshold for risk of future adverse events (Table 1), and thus GLOBE scores were not included in regression analyses.

## Results

### Thalamus

**Fig 1:**
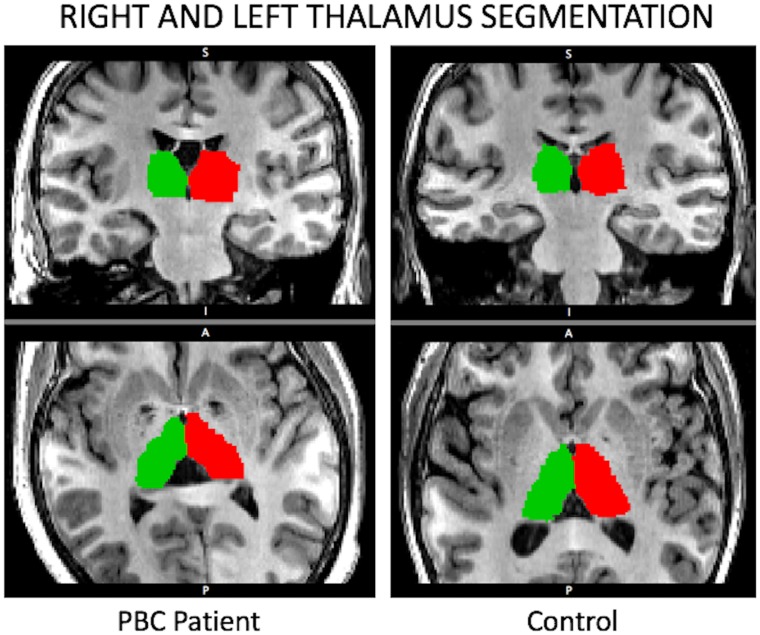
Example segmentations of the right (green) and left (red) thalamus from high-resolution anatomical T1-weighted MRI, for a PBC patient and a control subject.

**Fig 2:**
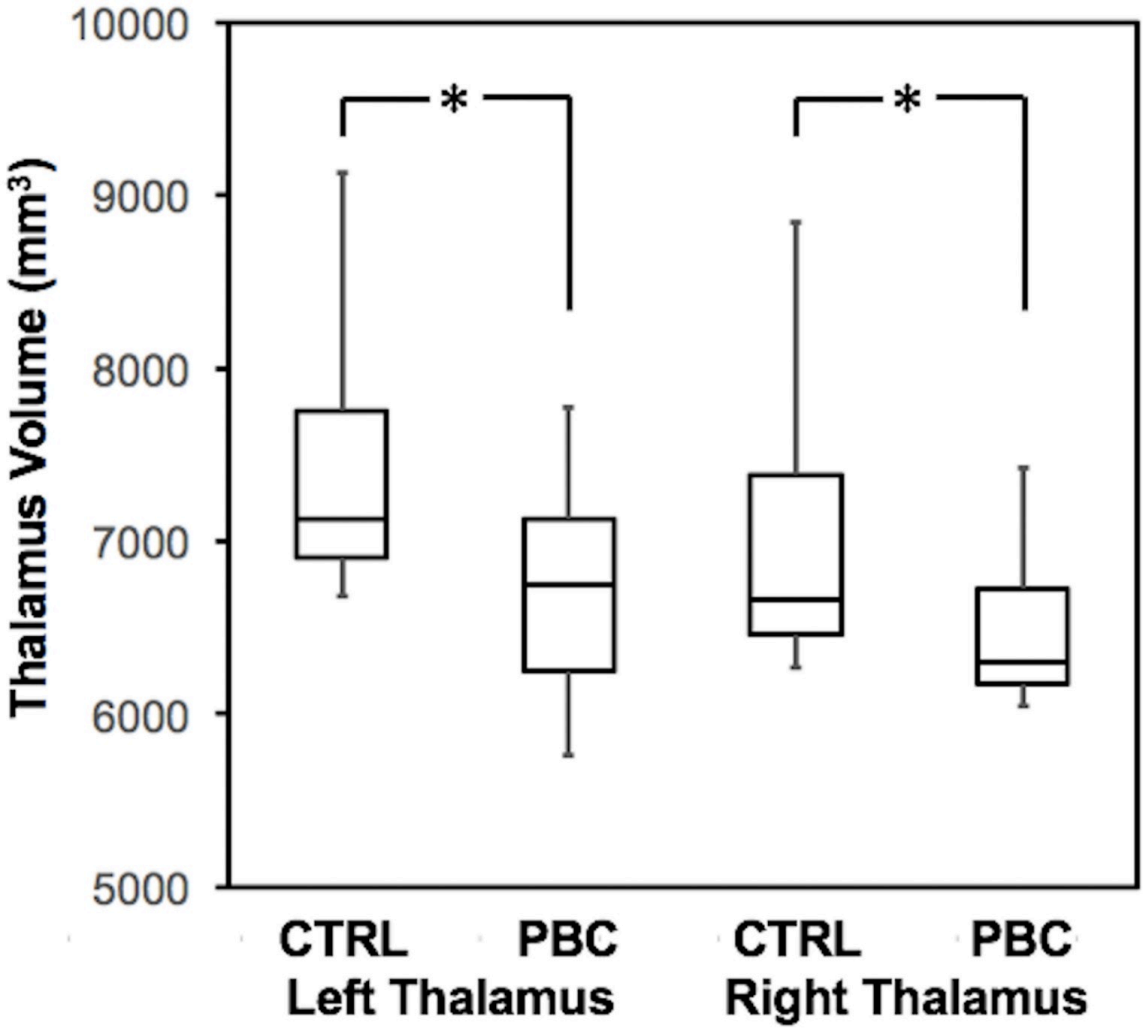
Thalamus volume is significantly reduced in both the left and right hemispheres in PBC patients compared to controls (*p < 0.03).

Successful thalamus segmentation was achieved for all participants. A *Freesurfer* segmentation of the thalamus for one PBC patient and one control subject are shown in Fig 1. PBC patients exhibited significantly reduced volume of the thalamus [F(1,27) = 8.87, *p* = 0.006]; follow-up *t*-tests demonstrated this was the case for both the left [*t*(27) = 2.96; *p* = 0.006] and right thalamus [*t*(27) = 2.35; *p* = 0.026] (Fig 2). Susceptibility of the thalamus did not differ between PBC patients and control subjects [F(1,27) = 0.22; *p* = 0.65], suggesting no presence of inflammation within the thalamus.

Thalamus volume and susceptibility were not associated with any of Fibroscan value, years since diagnosis, alkaline phosphatase level, UDCA response (Y/N) or total PBC-40 score. ALFF of the thalamus exhibited a strong trend toward a decrease in PBC patients relative to control subjects [F(1,28) = 3.80, *p* = 0.061], suggesting a potential reduction in the activity level of the thalamus.

**Fig 3:**
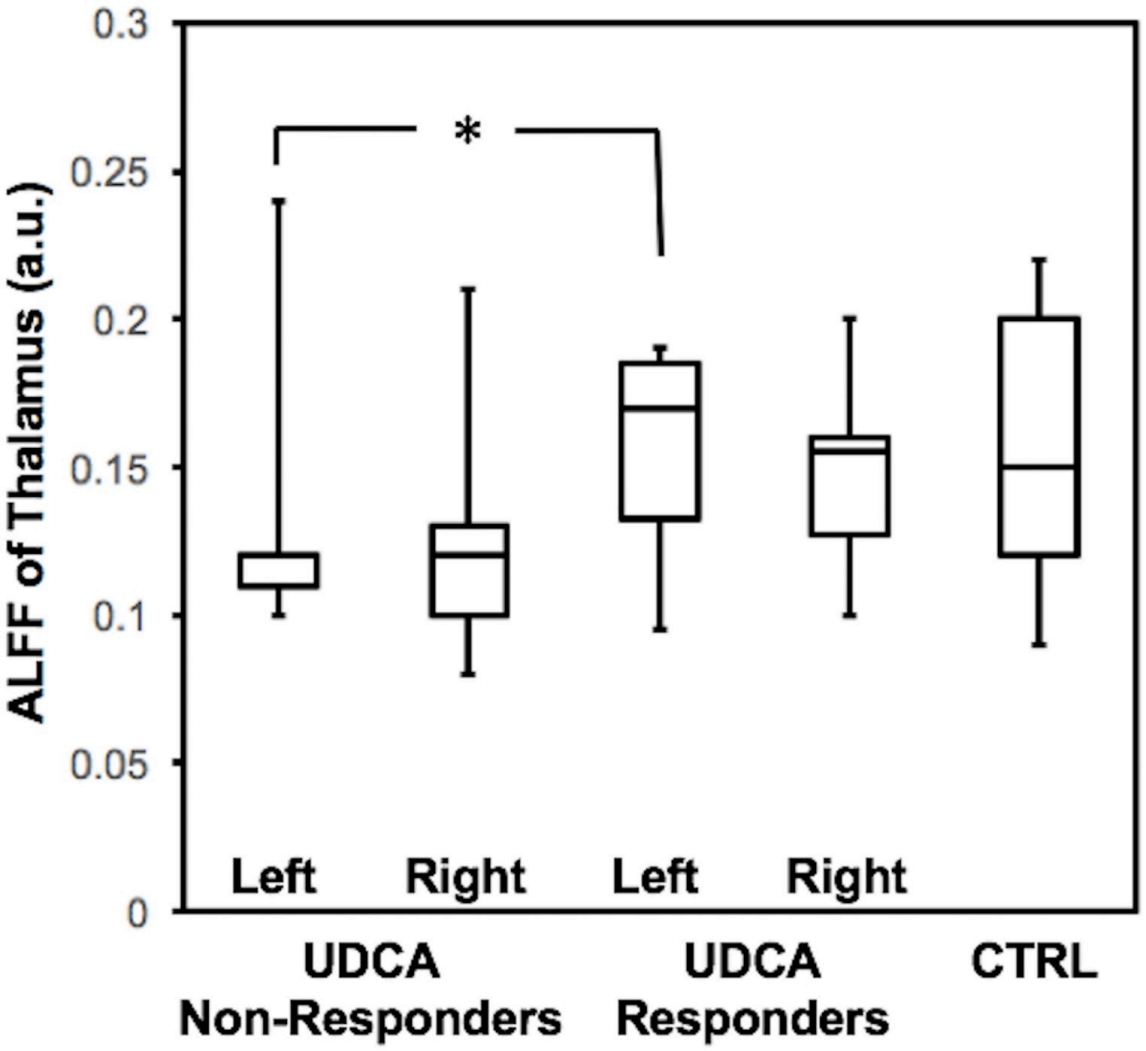
ALFF (in arbitrary units) of the left thalamus is significantly reduced in PBC patients who did not respond to UDCA therapy (*p* = .05), relative to UDCA responders, who were the same as control subjects (CTRL). ALFF of the right thalamus exhibited a strong trend (*p* = 0.055).

Linear regression analysis revealed that the combination of age and UDCA response significant predicted ALFF of the left [F(2,14) = 6.13, *p* = 0.015] and right thalamus [F(2,14) = 5.86, *p* = 0.017], and follow-up *t*-tests revealed that UDCA non-responders exhibited significantly lower left thalamus ALFF [*t*(12) = 2.18, *p* = 0.050] and near significantly lower right thalamus ALFF [*t*(12) = 2.12, *p* = 0.055] relative to UDCA responders, whose ALFF values were the same as control subjects (Fig 3). This suggests that PBC patients who do not respond to UDCA therapy exhibit reduced activity of the thalamus.

### Insula

**Fig 4:**
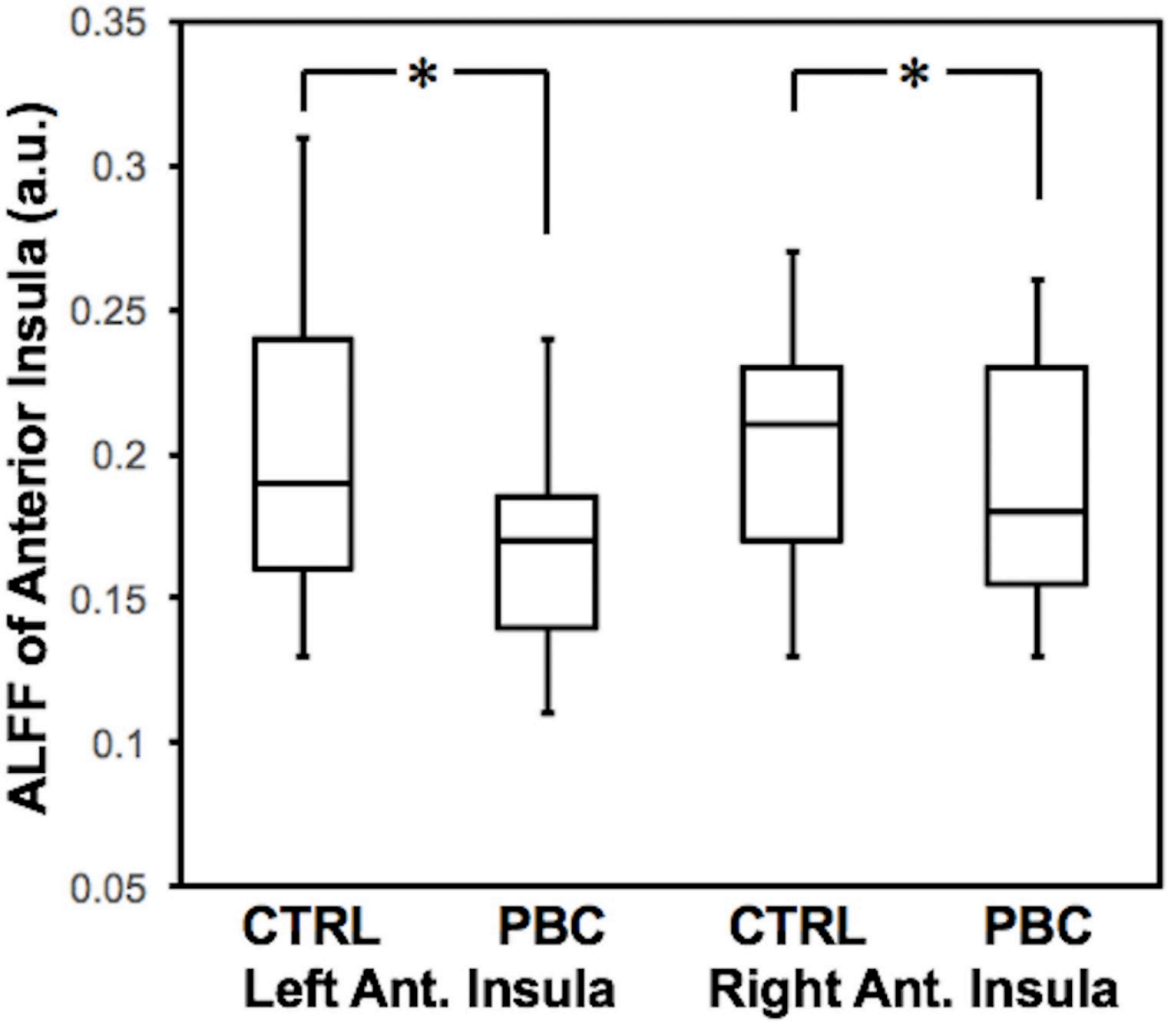
ALFF (in arbitrary units) of the anterior insula is reduced in both the left and right hemispheres in PBC patients compared to controls (*p < 0.02).

PBC patients exhibited significantly reduced ALFF of the anterior insula [F(1,28) = 7.16, *p* = 0.012]; follow-up *t*-tests demonstrated this was the case for both the left [*t*(28) = 2.48; *p* = 0.019] and right anterior insula [*t*(28) = 2.64; *p* = 0.013] (Fig 4). This suggests that PBC patients exhibit reduced activity of the anterior insula. Pruritus score was found to be a near-significant positive factor for anterior insula ALFF [F(1,28) = 4.08, *p* = 0.053], suggesting that reduced activity of the anterior insula may be driven by PBC patients who do not experience pruritus.

**Fig 5:**
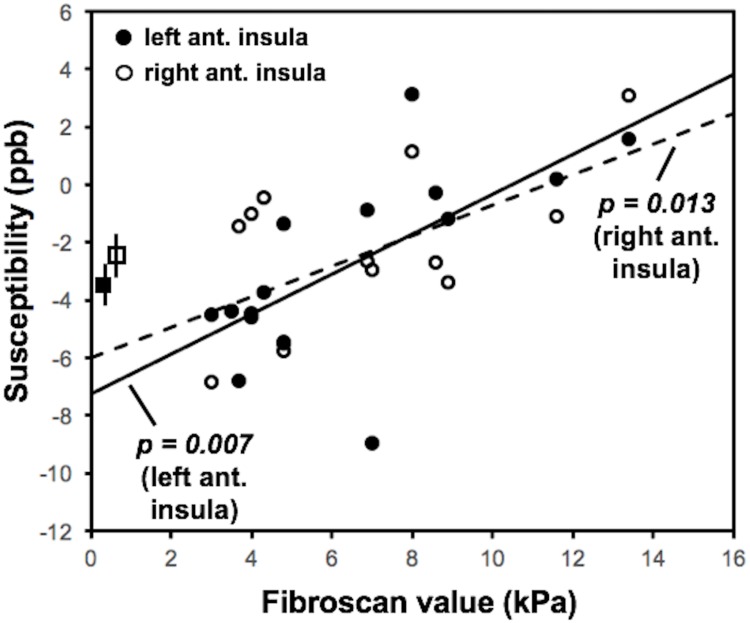
Fibroscan value is significantly associated with susceptibility (in parts per billion) within the left (*p* = 0.007) and right anterior insula (*p* = 0.013) of PBC patients. Mean susceptibilities of the left and right anterior insula for the control subjects are depicted by the square markers (error bars represent standard error of the mean).

There was no significant difference between PBC patients and control subjects in the susceptibility of the anterior [F(1,28) = 0.005; *p* = 0.95] or posterior insula [F(1,28) = 0.011; *p* = 0.92]. However, linear regression analysis revealed that Fibroscan value was a significant positive predictor of both left [*t*(14) = 3.19, *p* = 0.007] and right anterior insula susceptibility [*t*(14) = 2.86, *p* = 0.013] (Fig 5). That is, while there is no overall difference between PBC patients and control subjects in inflammation within the anterior insula, there is a suggestive link between Fibroscan value (i.e., liver stiffness) and indicators of inflammation.

## Discussion

Our results support the postulation that brain regions associated with interoception, specifically the thalamus and insula, exhibit altered structure and function in patients with PBC. Because the thalamus and insula are also known to be involved in a number of cognitive and affective functions, our findings also suggest there is a potential link between the altered state of interoception in PBC patients and the cognitive and mood deficits that are commonly reported. Although we cannot confirm the specificity of our findings to PBC, the fact that our patients have mild disease in the absence of cirrhosis and hepatic encephalopathy, and in the relative absence of behavioral symptoms, raises our confidence that the findings are indeed PBC-related.

Although the findings of the present study are highly novel, they need to be considered as preliminary, given the relatively low number of patients. Our patient numbers, in part, reflect the fact that we intentionally selected only patients with clinically mild disease to avoid potential issues associated with cirrhosis and hepatic encephalopathy; thus, the heterogeneity of our cohort was greatly reduced. Even in such a clinically mild disease group, PBC patients exhibit a 5–10% reduction in the volume of the thalamus, in the absence of neuroinflammation (i.e., no increase in susceptibility relative to controls). Moreover, years since diagnosis was not a significant predictor of volume reduction. These findings suggest that thalamic changes occur at an early stage in the disease course. The source of volume reduction is unclear. It is possible that during the early stages of PBC, thalamic volume reduction reflects the degradation of the supportive structure (e.g., neuropil) rather than a loss of neurons, and as a result, behavioral symptoms are not yet present. One could postulate that as the disease progresses, neurons eventually become lost, leading to symptom onset.

Given that the spontaneous activity of the thalamus of PBC patients who responded to UDCA therapy was the same as that of control subjects, thalamic changes may be reversible and amenable to interventions to decrease symptom burden. Although UDCA is not a neuroactive drug, our results clearly indicate it is capable of influencing brain function, further supporting the existence of pathways linking inflammatory responses of body organs with the brain. Whether the function of the thalamus is itself improved by UDCA, or if the activity of the thalamus increases due to the change in interoceptive signals from the body cannot be answered in the present study. Animal studies are warranted to elucidate the underlying mechanisms of the thalamus’ response to the normalization of liver biochemistry provided by UDCA. Further, longitudinal studies of patients pre- and post-UDCA therapy would substantiate whether UDCA indeed acts to normalize the activity of the thalamus.

The lack of a group difference in magnetic susceptibility of the thalamus may simply suggest that iron deposition within the thalamus is not associated with PBC, or at least that earlier stage disease is not characterized by an increase in iron deposition. However, our data suggests that liver fibrosis is associated with increased magnetic susceptibility of the anterior insula. Longitudinal studies involving patients with progressive disease could help to determine the relative timing of increases in fibrosis and insula susceptibility. PBC patients also exhibited reduced spontaneous activity of the anterior insula, and this was more evident in patients that did not experience pruritus (though it did not reach statistical significance). The anterior insula is thought to be heavily involved in interoception [41]. Task-related fMRI studies have shown that activation of the right anterior insula can predict accuracy in an interoceptive task, and volume is significantly correlated with interoceptive accuracy and awareness [42]. Our finding suggests there are aspects of PBC, potentially disease-associated immune-mediated inflammation within the liver, which may directly impact the anterior insula. While speculative, it may be that PBC acts to decrease the activity of anterior insula, but activity increases in response to the presence of pruritus. Studies that incorporate more direct interoceptive tasks in PBC patients with and without pruritus, could help to dissociate these potential influences on anterior insula activity.

No association with total PBC-40 score was found in any of our analyses; only the subscore pertaining to pruritus revealed an association with anterior insula activity, as mentioned above. It is possible that PBC patients with clinically mild disease simply do not exhibit a significant symptom burden to allow us to fully elucidate PBC-40 associated changes in structure and function of the thalamus and insula. A significant correlation between thalamic and insular structure and function and symptom/disease severity may exist in patients with later-stage disease. Future studies should aim to include a broader range of disease and symptom severities. However, what the present study does allow us to conclude is that even clinically mild PBC is associated with highly significant alterations in structure and/or function of the thalamus and insula.

Our study is the first of its kind to suggest that immune-mediated liver inflammation associated with PBC impacts the structure and function of the insula and thalamus. It also suggests that brain changes occur early in the disease course and may be reversible (e.g., UDCA normalizes thalamus activity in responders). Given the early onset of brain changes, widespread population screening may be warranted to therapeutically intervene before immune-mediated inflammation within the liver impacts brain structure and function, as for example, in patients who are AMA+ but show no biochemical evidence of cholestasis.
